# Cephalometric Sagittal Changes Suggestive of Maxillary Anterior Displacement and Mandibular Immediate Shift Following 3D-Guided Midpalatal Piezocorticotomy-Assisted MARPE in Adults: A Retrospective Cohort Study

**DOI:** 10.3390/jcm15114225

**Published:** 2026-05-29

**Authors:** Svitlana Koval, Daria Chepanova, Nika Stepanoff, Andrii Babii

**Affiliations:** 1 DrKoval Orthodontics, Private Practice, Boca Raton, FL 33341, USA; 2 Halmos College of Arts and Science, Nova Southeastern University, Fort Lauderdale, FL 33314, USA

**Keywords:** MARPE, adults, 3D-guided piezocorticotomy, 3D-GMP MARPE, mandibular rotation

## Abstract

**Objectives**: In this study, we aimed to evaluate the changes suggestive of maxillary anterior displacement in adults undergoing 3D-guided midpalatal piezocorticotomy-assisted Miniscrew-Assisted Rapid Palatal Expansion (MARPE), in addition to the contributing factors for forward maxillary movement and the subsequent immediate shift in the mandible. **Methods**: In this retrospective quasi-experimental study, cephalometric records of 80 adult patients (mean age 35.23 ± 8.76 years; 52 females and 28 males) were analyzed. Maxillary anterior displacement was assessed via SNA and A-Nperp(FH), while intermaxillary changes were measured using the ANB angle. Vertical and rotational changes were tracked through SN-MP, FH-MP, and various occlusal plane angles (OcP-FH, OcP-SN, OcP-GoMe). Facial height dimensions (TAFH, UAFH, LAFH, PFH) and dento-alveolar positions (U1-MP, U1LENGTH) were also recorded. **Results**: Following intervention, significant increases were observed in SNA (0.96°; 95% CI [0.48, 1.43]), ANB (1.42°; 95% CI [1.04, 1.80]), and A-Nperp(FH) (0.81 mm; 95% CI [0.24, 1.39]). The SN-GoMe angle increased by 0.98°, and Posterior Facial Height (PFH) decreased by 1.57 mm, while the upper incisor length (U1LENGTH) significantly decreased by 0.71 mm. **Conclusions**: In adults, 3D-guided midpalatal piezocorticotomy-assisted MARPE is associated with an increase in SNA, ANB, SN-GoMe, and A-Nperp(FH), and decreases in Posterior Facial Height (PFH) and the maxillary incisor length. The amount of mean midpalatal separation is moderately associated with the increase in SNA, while the increase in SNA is not associated with age or gender. Further 3D cephalometric studies would be beneficial to confirm the current findings.

## 1. Introduction

### 1.1. Background

Maxillary expansion has demonstrated positive effects for treating both Class II [1] cand Class III malocclusions [2] in growing individuals, and has been associated with an increase in pharyngeal airway volume [3] in growing individuals and improved quality of life in adults diagnosed with Obstructive Sleep Apnea (OSA) [4].

Maxillary forward movement is a desired outcome when treating both growing and non-growing individuals. A widely described treatment modality for late mixed dentition [5] in Class III malocclusion patients, the combination of a facemask and hybrid hyrax is considered, while for Class II growing individuals, where mandibular advancement is considered a treatment option, maxillary skeletal expansion is an adjunct, increasing its potential.

The Sella–Nasion–point A angle (SNA), referring to the point A distance to perpendicular to Nasion measurements, has been used in multiple previous studies as an indicator of sagittal maxillary changes [6,7,8,9] in patients treated with Surgically Assisted Rapid Palatal Expansion (SARPE) and the recently introduced Miniscrew-Assisted Rapid Palatal Expansion (MARPE).

Recently developed custom Miniscrew-Assisted Rapid Palatal Expansion devices have been proven to achieve predictable maxillary skeletal separation in adult patients, demonstrating fewer adverse effects and a high success rate compared to traditional SARPE, with multiple protocols introduced to increase this rate, including the slow-turn protocol (MASPE) [10], polycyclic turns [11], and 3D-guided midpalatal piezocorticotomy-assisted MARPE (3D-GMP MARPE) [12].

The minimally invasive nature of the piezosurgical intervention was described in previous studies [13] emphasizing the biological safety of the piezoelectricity and its significant tissue preservation compared to conventional techniques. Its effectiveness in orthognathic surgery was described in the study by AlAsseri and colleagues [14], while an innovative genioplasty technique in conjunction with the piezosurgical approach demonstrated promising results [13]. The 3D-guided approach to midpalatal piezocorticotomy was introduced by an author to preserve the nasal septum position and decrease the piezoelectrical incision (leaving the 8–10 mm of bone anterior to incisive foramen unseparated) while achieving successful and complete separation of the midpalatal suture in adult patients [12] with a low post-procedural pain level.

### 1.2. Objectives

In this study, we aim to evaluate the effects of maxillary anterior displacement associated with the 3D-GMP MARPE procedure alone, without a facemask or similar device, using lateral cephalometric measurements. The authors hypothesized that pre- and post-treatment lateral cephalometric measurements would show evidence of maxillary anterior displacement following the 3D-GMP MARPE protocol, while the mandible would undergo posterior rotation.

## 2. Methods

### 2.1. Study Design

Through this quasi-experimental retrospective cohort study, we evaluated the records of 80 patients who underwent the 3D-GMP MARPE procedure in the orthodontic practice of DrKoval Orthodontics located in Boca Raton, FL, USA. This study was conducted in accordance with the principles of the Declaration of Helsinki, and the study protocol was reviewed and approved by Solutions Institutional Review Board (Solutions IRB) (approval no. 0846/13 January 2026). Written informed consent was obtained from all participants, and the STROBE reporting guidelines were followed.

### 2.2. Participants

**Figure 1:**
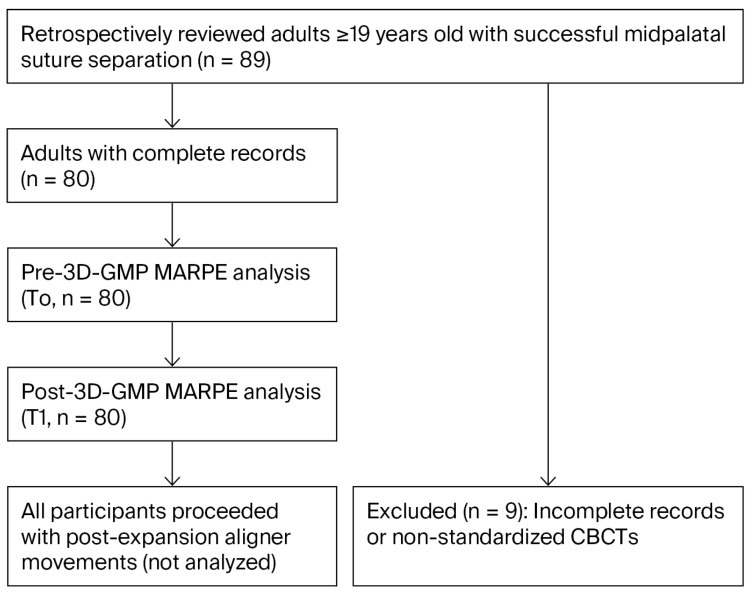
The 3D-GMP MARPE flowchart diagram.

A total of 89 participants entered the study, and after initial review, the records of 80 participants with a mean age of 35.23 ± 8.76 years were evaluated. Inclusion criteria included adults aged 19 years and older with skeletally narrow maxilla; uni- or bilateral posterior crossbite; Skeletal Class I, II, or III malocclusion; and no history of maxillary skeletal expansion, orthognathic surgery, connective tissue diseases, autoimmune conditions, or chemotherapy. The study sample consisted of practice patients initially screened for sleep-disordered breathing (SDB), and exhibiting signs of daytime fatigue, fragmented sleep, increased NOSE scores, moderate-to-high Epworth scores, and minor Temporomandibular disorders evaluated with the DC-TMD scale and clinical examination. The study population included 52 females (65%) and 28 (35%) males who underwent the 3D-GMP MARPE procedure as part of the initially recommended treatment. The descriptive characteristics of the study population are included in Table 1, and the flowchart diagram is available in Figure 1.

### 2.3. Data Sources/Measurements

Lateral cephalometric radiographs were rendered from the CBCT volumes of each patient. The CBCT images were obtained with Planmeca G7 Viso (FOV 022 × 021 cm, Planmeca, Helsinki, Finland) with an Ultra-Low-Dose protocol (ULD), 100 kV, 6.3 msA, 30 mkmSv, and 600 voxel size. The exported lateral cephalometric radiographs processed in Romexis (Planmeca, Helsinki, Finland) were further traced manually in WebCeph (Seongnam-si, Republic of Korea) by the Principal Investigator (SK). To evaluate measurement reliability across individual parameters, a variable-specific analysis was conducted. The intraclass correlation coefficient (ICC), along with Dahlberg’s formula for method error (ME), was calculated independently for each primary and secondary variable. The analysis yielded high relative and absolute reliability across all landmarks. Specifically, the cephalometric angular metrics demonstrated strong stability: SNA presented an ICC of 0.94 (95% CI: 0.89–0.97) with a Dahlberg error of 0.21 degrees; ANB showed an ICC of 0.95 (95% CI: 0.91–0.98) with an ME of 0.15 degrees; and SN-GoMe exhibited an ICC of 0.92 (95% CI: 0.86–0.96) with an ME of 0.34 degrees. Similarly, linear and structural metrics demonstrated high reproducibility: A-Nperp(FH) displayed an ICC of 0.91 (95% CI: 0.85–0.95) and a method error of 0.18 mm; PFH showed an ICC of 0.93 (95% CI: 0.88–0.96) with an ME of 0.28 mm; and U1LENGTH demonstrated an ICC of 0.90 (95% CI: 0.84–0.94) with an ME of 0.22 mm.

### 2.4. Variables

Fifteen measurements were conducted for the purposes of this study (Table 2). Maxillary sagittal changes were evaluated using angular (SNA) and linear measurements (A-Nperp(FH)), and the intermaxillary relationship was measured using the angular ANB measurement. Angles SN-MP and FH-MP were used to evaluate the direction and amount of maxillary plane rotation, while occlusal plane rotation and cant were measured with OcP-FH, OcP-SN, and OcP-GoMe angles. Linear facial height measurements were evaluated with TAFH (total anterior facial height, NaMe), UAFH (upper anterior facial height, Na-ANS), LAFH (lower anterior facial height, ANS-Me), and PFH (Posterior Facial Height, S-Go). Dento-alveolar measurements of the maxillary incisor positions included angular U1-MP and linear U1LENGTH measurement.

### 2.5. Bias

CBCT images taken with an open-mouth posture were eliminated from this study; instead, the CBCT volumes imaged in a standing position with no head or chin support were evaluated. Operator and measurement bias were addressed with a combination of artificial intelligence, cephalometric landmarking, and repeated measurements. Patient selection biases could not be avoided due to the retrospective study design, the study sample being limited to patients of a single private practice, and a combination of anatomical characteristics compatible with the SDB phenotype. Bias related to our pending patent was minimized due to the double-blind data management and statistical analysis technique.

The SNA and A-Nperp(FH) metrics were derived from intracranial landmarks independent of external reference axes, rendering them immune to variations in cervical extension or flexion. Conversely, lateral head tilt presented a potential source of error by inducing dual contours of bilateral structures. To control for this rotational artifact, head orientation was standardized within the Romexis software (Version 6.4.7.129) to achieve optimal superimposition of contralateral anatomical landmarks. Confounding from dentoalveolar remodeling was systematically ruled out based on three parameters: (1) the absence of concurrent orthodontic or dental arch interventions, (2) a study protocol designed to isolate immediate post-maxillary distraction changes, and (3) the prevention of secondary passive tooth migration or physiological drifting, achieved by acquiring cone-beam computed tomography (CBCT) scans immediately upon the cessation of active expansion.

### 2.6. Study Size

A sample of 80 participants who met the inclusion criteria was enrolled in this study. To confirm the adequacy of this sample size, a retrospective power analysis for a paired *t*-test was conducted (alpha = 0.05, power = 0.80, SD = 2.12). The analysis indicated that a minimum of 41 participants would be required to detect a clinically meaningful difference for the SNA parameter (primary outcome). Thus, the actual sample size of 80 participants provided more than sufficient statistical power to detect relevant treatment changes. For the secondary outcome of A-Nperp(FH), a post hoc power analysis was conducted to evaluate the sensitivity of the utilized sample size (N = 80). Based on a pre-to-post mean change from 0.03 ± 2.57 mm to 0.84 ± 2.57 mm (representing a mean difference of 0.81 mm and an effect size delta of 0.315), a sample size of 80 participants provided an estimated statistical power of 79.5% (approximately 80%) at a two-tailed significance level (alpha) of 0.05. This demonstrates that the study was adequately powered to detect subtle variations in sagittal maxillary position.

### 2.7. Intervention

**Figure 2:**
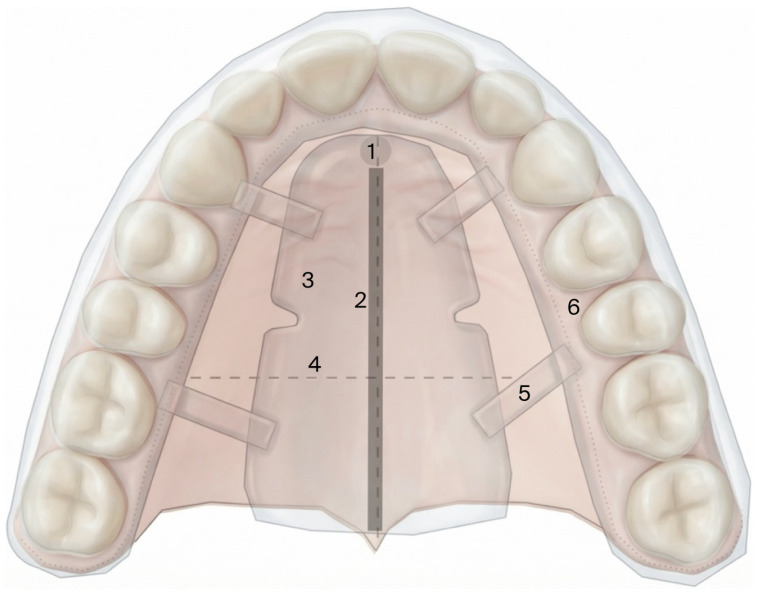
Schematic image of the 3D-printed midpalatal piezocorticotomy guide: 1—incisive foramen; 2—incision notch for the midpalatal piezocorticotomy; 3—guide base covering the hard palate; 4—location of the transverse palatal suture; 5—3D-printed guide connector; 6—3D-printed guide occlusal splint covering maxillary teeth occlusal surfaces. The dash lines represent midplatal suture (vertical) and transverse suture (horizontal).

**Figure 3:**
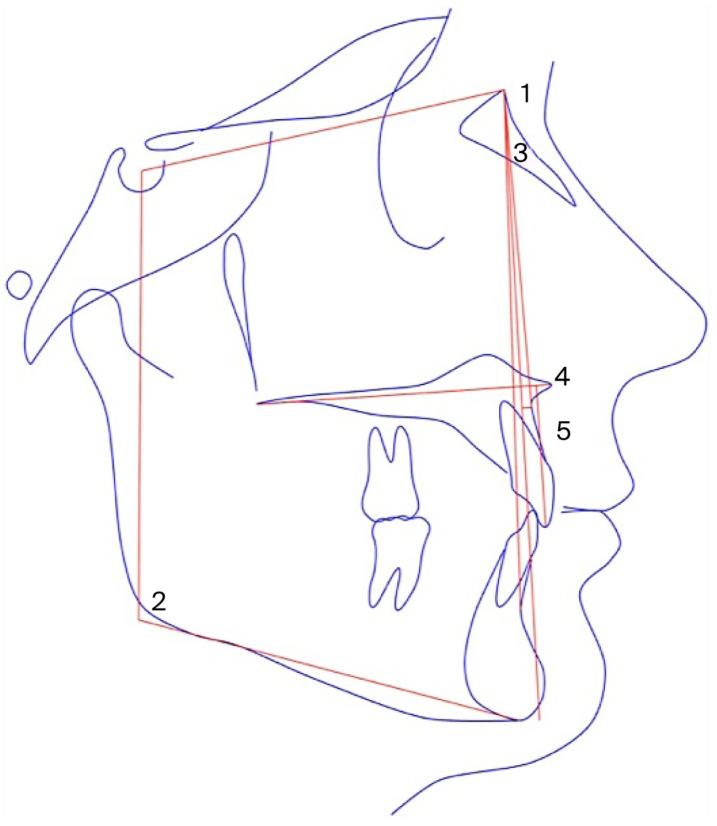
Lateral cephalometric tracing and measurements used for pre- and post-treatment analysis: 1—SNA; 2—Posterior Facial Height (PFH); 3—ANB; 4—A-N perp(FH); 5—U1LENGTH.

The 3D guide for the midpalatal piezocorticotomy (Figure 2) was fabricated based on the initial CBCT volume merged with the iTero intraoral scans (iTero, Align Technology, San Jose, CA, USA). The piezocorticotomy guide was used to precisely place the piezocorticotomy cuts, followed by the custom MARPE placement (Figure 3). The three-dimensional (3D)-guided midpalatal piezocorticitomy was executed via a flapless approach under local anesthesia, utilizing either 3% lidocaine with 1:100,000 epinephrine or 0.5% bupivacaine with 1:200,000 epinephrine. Corticotomy incisions were extended to the precise depths predetermined during the 3D-planning phase, as dictated by the custom piezoguide. The osteotomies were performed using a piezoelectric handpiece under continuous irrigation with a sterile physiological saline solution. Following the predetermined notches of the piezoguide, the incisions spanned linearly from the distal boundary of the incisive canal to the posterior nasal spine (PNS). The post-operative pain management protocol included the use of Acetaminophen 500 mg (max daily dose not exceeding 3000 mg) and Ibuprofen 800 mg (maximum daily dose not exceeding 3200 mg). Patients were instructed to take antibiotic premedication 1 h before the procedure (Amoxicillin 2 × 500 mg) and continue with the antibiotic for the next 5 days after the procedure. Alternative antibiotics were prescribed when sensitivity or prior reactions to Penicillin antibiotics were reported. The procedure was deemed successful upon manual perception of penetration of both cortical plates of the nasal base and the absence of adverse events (bleeding from the intervention site, nasal bleeding, acute pain).

**Figure 4:**
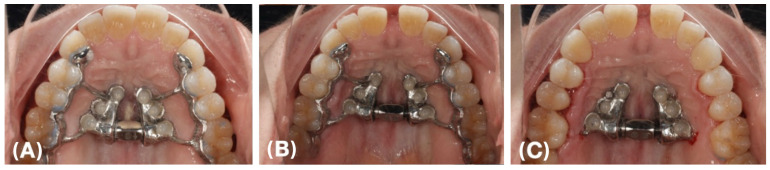
Design of the custom MARPE used: (**A**) after 3D-guided midpalatal piezocorticotomy and installation (TAD screwheads are covered with composite); (**B**) 6 weeks into expansion; (**C**) upon completed expansion, with tooth-anchored parts of the milled framework removed.

**Figure 5:**
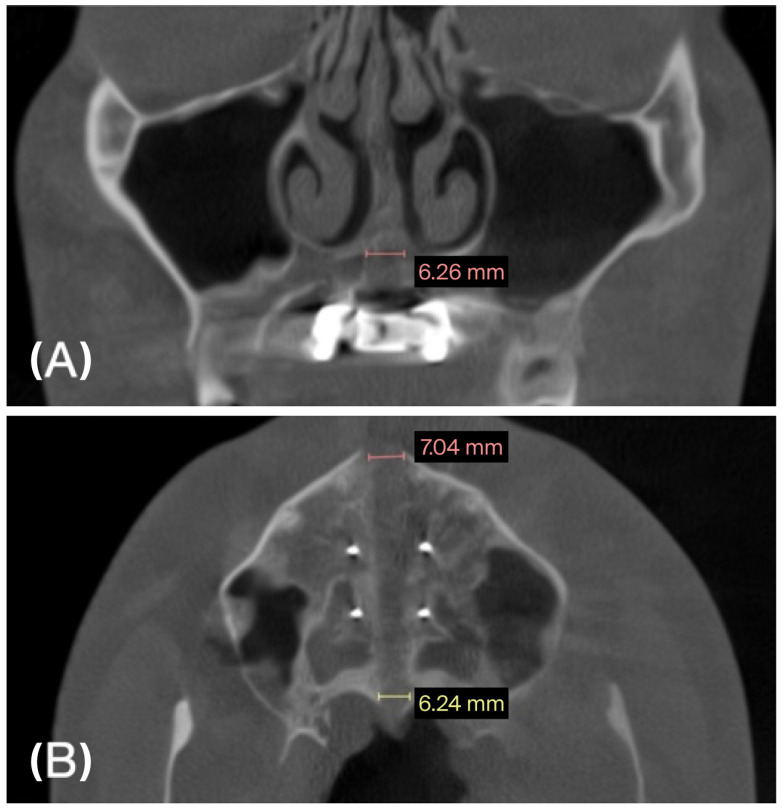
Resulting midpalatal suture separation: (**A**) coronal view at the maxillary first molar level, indicating 6.26 mm of separation at the nasal base (ANS-PNS plane); (**B**) axial view of the same patient demonstrating 7.04 mm of midpalatal separation at the ANS and 6.24 mm at the PNS (89% parallelism).

The MARPE activation was initiated 7 days after its placement, following the healing stage. Further activation was kept at one turn a day (0.11 mm) for both males and females after the midpalatal split was confirmed. Powerscrew activation (TigerDental, Hörbranz, Austria) was 0.11 mm per turn. Complete (from ANS to PNS) midpalatal suture separation was then confirmed with CBCT (Figure 4 and Figure 5). The average expansion stage (T0-T1) lasted for 8–10 weeks, with the midpalatal split occurring between day 14 and day 21.

### 2.8. Statistical Methods

All statistical analyses were conducted in StataNow/SE 19.5 (Stata Corp, College Station, TX, USA). Descriptive statistics were used to summarize the studied population. To test the data normality, the Shapiro–Wilk test and quantile–quantile plots were used for every measured variable. Paired and two-sample *t*-tests were used to identify differences between groups for all measured variables. To demonstrate statistical significance, a *p*-value of <0.05 was used.

## 3. Results

### 3.1. Participants

All 80 participants demonstrated successful midpalatal suture separation. The mean anterior separation achieved was 5.55 ± 1.34 mm in females, 6.65 ± 1.95 mm in males, and 5.94 ± 1.65 mm in the overall sample. No severe or major adverse events were recorded within the study cohort. Postoperative pain was transient and adequately managed via standard over-the-counter analgesics within the first 3 to 5 days. There were no incidents of prolonged post-surgical hemorrhage, localized infection, or mucosal impingement beneath the appliance components. From a technical and anatomical standpoint, all surgical piezoguides demonstrated accurate intraoperative seating with zero instances of clinical misfit. Temporary anchorage device (TAD) stability remained uncompromised, with no secondary loosening or failure noted during active distraction or retention. Furthermore, radiographic evaluation revealed no structural root proximity concerns, adverse periodontal bone loss, or unplanned skeletal or dentoalveolar complications across the 80-patient sample.

### 3.2. Descriptive Data

To protect against type I error inflation from multiple comparisons, a strict hierarchy of endpoints was established. The primary skeletal outcome of this investigation was defined as the change in the SNA. A priori power calculations confirmed that the sample size (N = 80) was robustly configured to detect alterations in this primary endpoint with a statistical power of 80% and a significance level (alpha) of 0.05.

The linear measurement A-Nperp(FH) (mm) was designated as the principal secondary outcome, for which the study sample yielded an independent achieved power of 79.5%. All remaining skeletal, positional, and dentoalveolar variables were categorized as standard secondary outcomes.

Because no formal alpha-level multiplicity adjustments (such as a Bonferroni correction) were applied to the secondary metrics, *p*-values derived from these secondary comparisons should be interpreted with caution. Consequently, all subsequent stratification, subgroup analyses, and percentile-based cohort findings were treated as purely exploratory, serving to generate hypotheses for future prospective validation rather than to establish definitive confirmatory conclusions.

Among the 15 measurements compared between the pre- (T0) and post-expansion (T1) stages, only six showed a significant change (SNA, ANB, A-Nperp(FH), SN-GoMe, PFH, and U1LENGTH). The intraclass correlation coefficient (ICC) and Dahlberg’s formula for method error (ME) were calculated independently for each primary and secondary variable. The analysis yielded high relative and absolute reliability across all landmarks. Specifically, the cephalometric angular metrics demonstrated strong stability: SNA presented an ICC of 0.94 (95% CI: 0.89–0.97) with a Dahlberg error of 0.21 degrees; ANB showed an ICC of 0.95 (95% CI: 0.91–0.98) with an ME of 0.15 degrees; and SN-GoMe exhibited an ICC of 0.92 (95% CI: 0.86–0.96) with an ME of 0.34 degrees. Similarly, linear and structural metrics demonstrated high reproducibility: A-Nperp(FH) displayed an ICC of 0.91 (95% CI: 0.85–0.95) and a method error of 0.18 mm; PFH showed an ICC of 0.93 (95% CI: 0.88–0.96) with an ME of 0.28 mm; and U1LENGTH demonstrated an ICC of 0.90 (95% CI: 0.84–0.94) with an ME of 0.22 mm.

Parameters measuring the inclination of the maxillary plane relative to the Frankfort horizontal (FH-MP) or base of the skull (SN-MP) did not show a significant change, indicating no significant maxillary plane rotation/inclination with the procedure. Occlusal plane inclination (OcP-FH, OcP-SN, OcP-GoMe) did not significantly change with the intervention either.

### 3.3. Outcome Data

SNA significantly increased by 0.96 degrees with a 95% CI of [0.48, 1.43] following intervention, ANB by 1.42 degrees (95% CI [1.04,1.80]), A-Nperp(FH) by 0.81 mm (95% CI [0.24,1.39]), and SN-GoMe angle by 0.98 degrees (95% CI [0.42, 1.54]), and PFH (Posterior Facial Height) significantly decreased by 1.57 mm (95% CI [−2.82, −0.32]) and upper incisor length (U1LENGTH) by 0.71 mm (95% CI [−1.36, −0.05]).

### 3.4. Other Analyses

**Figure 6:**
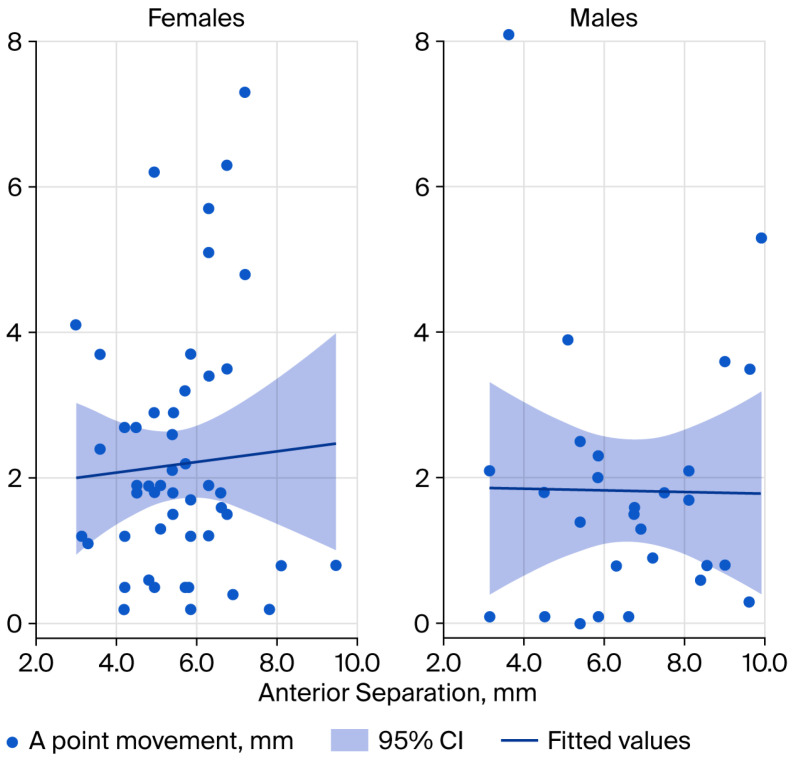
Scatter plot with fitted values and 95% CI of the A-point movement measured as an absolute change in A-Nperp(FH) by gender, indicating an increase in forward movement in females with the increase in ANS separation, and a slight decrease in the A-point movement in males as ANS separation increased.

**Figure 7:**
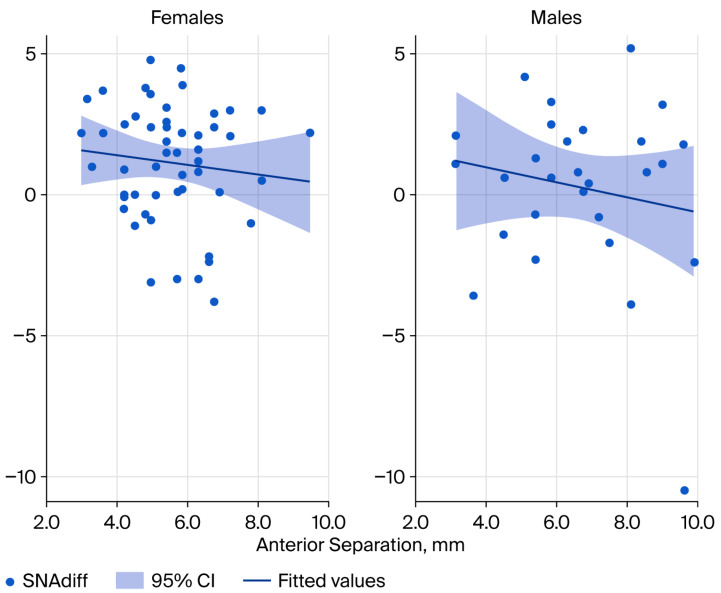
SNA difference with the amount of anterior midpalatal separation determined by gender. The downward slope of both fitted lines indicates the decrease in the SNA growth with increasing anterior separation of the midpalatal suture.

Interpercentile group analysis of all 15 variables demonstrated significant differences between percentile groups for A-Nperp(FH). While the Kruskal–Wallis test indicated a significant difference in A-N Perp (*p* = 0.04), the small group sizes (N = 20) suggested that the study may have been underpowered to detect subtle intergroup variations consistently. Angle classification group analysis was performed between Class I and Class II groups with significant intergroup OcP-FH variable differences, while the Class I group individually demonstrated significant differences in SNA, ANB, SN-GoMe, OcP-SN, and OcP-FH variables; these differences were significant and could likely be replicated. The Skeletal Class II group showed significant differences in SN-GoMe, A-Nperp(FH), and PFH variables. Scatter plots (Figure 6 and Figure 7) with fitted SNA and A-Nperp(FH) increase values demonstrated a steady pattern in A-point forward movement, with expansion at an average level of 2 mm for males and increasing point A displacement with expansion for females.

To isolate the independent predictors of maxillary skeletal change and control for clinical confounding, a multivariable linear regression analysis was performed with the change in SNA (DiffSNA) designated as the dependent variable. The regression model factored in age, sex, baseline sagittal severity (ANB), baseline vertical craniofacial configuration (SN-GoMe, SNMP, PPA, OcPSN), and the net magnitude of distraction adjustment.

The combination of these predictors significantly accounted for the variation in post-treatment maxillary movement (F(9, 70) = 3.46, *p* = 0.0014), demonstrating a model coefficient of determination (R^2^) of 0.3081 and an adjusted R^2^ of 0.2192. Among the evaluated covariates, baseline sagittal jaw relationship (ANB) emerged as a highly significant independent predictor (beta = 0.36, 95%CI: 0.151 to 0.573, *p* = 0.001), indicating that patients presenting with more severe initial sagittal discrepancies experienced a greater magnitude of forward maxillary change.

Additionally, the overall magnitude of expansion (MEANSEP) demonstrated a strong positive trend that closely neared statistical significance (beta = 0.348, 95% CI: −0.002 to 0.699, *p* = 0.052). Chronological age (*p* = 0.555), sex (*p* = 0.489), and baseline vertical divergence parameters (*p* > 0.05) did not act as statistically significant independent drivers of the immediate post-distraction DiffSNA changes in this cohort (Table 3, Table 4 and Table 5).

Subgroup analysis based on skeletal classification is presented in Table 6 and Table 7.

## 4. Discussion

### 4.1. Key Results

In the current study, we showed a significant increase in the anterior position of the maxillary base when measured on lateral cephalometric radiographs. The measurements used included SNA and A-Nperp(FH), both using point A as a reference for the most anterior point of the maxillary base. The average increase in SNA was 0.96 degrees, with a 0.81 mm increase in A-Nperp(FH) in the study sample of 80 subjects and 5.94 ± 1.65 mm of anterior midpalatal suture separation. The increases in point A-associated measurements (SNA and A-Nperp(FH)) following treatment were concomitant with the significant decrease in the U1-LENTH measurement. The upper incisor length projection was measured from the ANS-PNS plane to the incisal edge of the maxillary central incisor, and its decrease could have contributed to the respective tooth’s buccal tipping with palatal inclination of the root (thus, movement away from the buccal cortical plate). No incisor movements were performed with active tooth-borne appliances while expanding the maxilla; thus, the effects of point A anterior displacement could be attributed to the maxillary base displacement rather than dento-alveolar effects.

Using point A as a sagittal reference for the maxillary base position has been justified by multiple earlier studies, including those evaluating the effects of RPE [8], SARPE [6,9], and MARPE alone [15]. An evaluation of the maxillary sagittal changes after Surgically Assisted Rapid Palatal Expansion (SARPE) was published by Jia-Hong Lin in a systematic review and meta-analysis [7], reporting a 0.50° ± 0.08° increase in SNA.

To better understand the clinical and anatomical factors influencing immediate skeletal outcomes, a multivariable linear regression model was constructed to control for confounding variables. Covariates, including age, sex, initial sagittal severity (ANB), baseline vertical craniofacial configurations, and the net magnitude of mechanical expansion, were intentionally selected based on their documented roles in conventional maxillary expansion and distraction osteogenesis. The model successfully explained approximately 30.8% of the variance observed in the primary outcome (R^2^ = 0.3081, *p* = 0.0014).

A principal finding from this adjusted analysis was the highly significant independent effect of the baseline sagittal jaw relationship (ANB; beta = 0.362, *p* = 0.001). This indicates that patients presenting with a more severe initial Class III sagittal discrepancy or lower initial ANB value experienced a greater relative magnitude of forward maxillary change (DiffSNA). Clinically, this suggests that the 3D-guided piezocorticitomy effectively releases structural resistance along the midpalatal suture, allowing the distraction forces to efficiently advance the maxilla, particularly in individuals with severe anteroposterior deficiencies who exhibit high structural indications for skeletal modification.

Furthermore, the net magnitude of mechanical separation (MEANSEP) demonstrated a strong positive trend that closely neared the threshold of statistical significance (beta = 0.348, *p* = 0.052). This near-significant threshold highlights a direct dose–response relationship, indicating that greater absolute activation of the expansion appliance yields a corresponding volumetric and spatial advancement of point A.

Conversely, chronological age (*p* = 0.555) and baseline vertical divergence parameters—such as SN-GoMe (*p* = 0.203) and SNMP (*p* = 0.114)—did not emerge as statistically significant drivers of DiffSNA.

In the current study, we evaluated the sagittal changes suggesting maxillary forward displacement between two skeletal classes (I and II). While the pre- and post-expansion difference in Skeletal Class I was significant, Skeletal Class II did not show significant changes. This may be attributed to a higher proportion of downward maxillary rotation in Skeletal Class II patients compared to Skeletal Class I. This is in agreement with the results outlined in Table 7, emphasizing the significant decrease in PFH and increase in mandibular plane angle (SNGoMe) in Skeletal Class II patients. In addition to the results of the interpercentile group analysis, these findings lie within the exploratory plane and would require larger sample sizes to be studied further.

Maxillary sagittal changes resulting from the expansion technique remain controversial in adults; nevertheless, the midpalatal disruption potential may contribute to the circum-maxillary distraction, with possible advancement of the maxillary base in adults. This effect has been explored in the present study. Previously, Skeletal Class III patients have been the focus of maxillary forward movement-oriented treatment using a combination of facemask therapy and various expansion techniques.

The authors of several earlier studies reported clockwise maxillary rotation with SARPE [7,9]. We did not confirm maxillary downward rotation with 3D-GMP MARPE, therefore showing no statistically significant difference between pre- and post-expansion measurements. Iodice and co-authors [16] did not find any statistically significant changes in the sagittal maxillary base position with SARPE, only reporting maxillary incisor inclination change. Our study results are in agreement with this finding, reporting dento-alveolar change in the maxillary incisor vertical position (statistically significant decrease in maxillary incisor length) with intervention. Xue and co-authors [17] reported that SNA and ANB increase with MARPE treatment in adults (0.54 degrees of increase for SNA and 1.54 degrees of increase for the ANB angle), while our study showed an ANB increase of 1.42 degrees. Thuy and Trang [18] reported no change to the Maxillary Plane Angle and minimal changes to the SNA and ANB angles with MARPE intervention.

McMullen and colleagues evaluated maxillary position changes in growing and non-growing patients treated with MARPE, concluding that there is greater downward displacement of ANS in growing patients [19]. Sayar [20] and colleagues evaluated growing and non-growing patients treated with Rapid Palatal Expansion (RPE) devices and found no difference between groups in terms of skeletal and dental outcomes of RPE.

The facemask–hybrid hyrax combination treatment produced 1.87 ± 1.06 degrees of increase in SNA [21] and 3.04 mm of point A forward movement after 10 months of protraction time in growing individuals [22]. Tarraf and colleagues reported 4.26 ± 2.15 degrees of SNA increase with a combined hybrid hyrax and mandibular miniplate treatment in growing subjects [23]. Our data shows that adult maxillary skeletal expansion with 3D-GMP MARPE produces, on average, 0.96 ± 2.12 degrees of SNA increase in a mixed sample of Skeletal Class I, II, and III subjects with various baseline rotations of the maxillary plane and a mixed pattern of midpalatal suture separation (with and without palatine bone distraction).

Factors such as age, sex, and suture maturation have been evaluated as contributing to the success of maxillary skeletal expansion [24]. Chen and Kapetanovic [25] confirmed that for adult patients, the success of midpalatal suture separation is not related to age and gender, while Shin [26] and colleagues found a negative correlation between the amount of midpalatal suture separation and a subject’s age and sex. These varying outcomes are related to the difference in techniques used (SARPE, MARPE, RPE), the amount of midpalatal separation achieved, and study group characteristics. The 3D-GMP MARPE technique has proven to be successful irrespective of the midpalatal suture maturation stage and patient age or gender, as shown in previous case reports and case series [12,27,28]. The anterior separation amounts evaluated in this study averaged 5.94 ± 1.95 mm, which is higher than the frequently reported midpalatal separation amounts [15,29,30]. Additionally, 3D-GMP MARPE did not produce any significant discomfort throughout the expansion stage, which is in accordance with the study by Elshehaby [31], who reported lower pain levels when micro-osteoperforations of the midpalatal suture were combined with MARPE treatment.

Scatter plots (Figure 5 and Figure 6) show a visual representation of the relationship between the anterior separation amount and the increase in both SNA and A-Nperp(FH), but linear regression analysis did not confirm a statistically significant association between the two, while indicating that the mean midpalatal separation, rather than anterior alone, is directly related to the point A position change with 3D-GMP MARPE. (Table 8).

The decrease in Posterior Facial Height reported in the current study was statistically significant for the total sample with intervention, indicating an immediate mandibular response to maxillary skeletal expansion. SN-GoMe angle increase was concomitant with the ANB increase, indicating posterior mandibular rotation.

It is important to acknowledge that while the 3D-guided surgical protocol offers distinct clinical advantages, a formal economic evaluation was not performed in the present study. Consequently, definitive conclusions regarding its cost-effectiveness cannot be drawn. Future prospective, comparative investigations are warranted to systematically evaluate procedure costs, clinical chair time, active surgical duration, and advanced imaging requirements alongside complication rates and patient-reported outcome measures.

### 4.2. Limitations

The limitations of the current study included analyzing a restricted sample of patients with pre-existing symptoms of sleep-disordered breathing. The predominant tendency towards having higher ANB angles before treatment and the smaller number of patients with a Skeletal Class III base relationship did not allow for the comparison of all skeletal classes. The 2D nature of the lateral cephalometric measurements was an additional limiting factor, while further studies would benefit from 3D evaluation of the maxillary landmark anterior displacement. The study design was limited to a single-arm retrospective analysis with no comparison group.

### 4.3. Interpretation

Our current data needs to be carefully interpreted due to the retrospective study design with no comparison group. It is unclear whether the observed anterior displacement of point A is attributed to the combination technique (3D-GMP MARPE) or is due to the sample characteristics. The age and sex were eliminated as confounders of the success of midpalatal separation, while further studies need to explore additional confounders and/or effect modifiers of sagittal maxillary displacement.

### 4.4. Generalizability

The reported data have limited generalizability due to the nature of the study sample. Further studies are recommended to increase the generalizability of the current findings.

## 5. Conclusions

The 3D-guided midpalatal piezocorticotomy-assisted MARPE technique in adults is associated with an increase in SNA, ANB, SN-GoMe, and A-Nperp(FH) and a decrease in Posterior Facial Height and maxillary incisor length. The amount of mean midpalatal separation is moderately associated with the increase in SNA, while the increase in SNA is not associated with age or gender. Further 3D cephalometric studies would be beneficial to confirm the current findings.

## Figures and Tables

**Table 1:** Characteristics of the study participants. Mean values and standard deviations are presented for each continuous variable.

Characteristics	Gender		
	Females	Males	Total
Number	52 (65%)	28 (35%)	80 (100%)
Age	37.94 (7.71)	30.18 (8.45)	35.23 (8.76)
Anterior midpalatal separation, mm	5.55 (1.34)	6.65 (1.95)	5.94 (1.65)
ANB before Tx, degrees	4.07 (2.34)	3.12 (2.99)	3.74 (2.61)
SNA before Tx, degrees	80.59 (4.22)	81.26 (3.38)	80.82 (3.94)
SN-ANSPNS before Tx, degrees	8.31 (3.35)	7.73 (3.85)	8.11 (3.52)

**Table 2:** Descriptions of the parameters measured in the study.

ANTERSEP	Separation of the midpalatal suture at the level of ANS, mm
SKCLASS	Skeletal Class
SNA	SNA before treatment, degrees
ANB	ANB angle, degrees
A-Nperp(FH)	Distance from point A to the perpendicular-to-FH point N, mm
SN-MP	SN-ANS-PNS angle before treatment, degrees
FH-MP (PPA)	Palatal Plane to FH angle, degrees
SN-GoMe	Angle between the SN and GoMe, degrees
OcP-SN	Angle between the SN plane and the occlusal plane, degrees
OcP-GoMe	Angle between the GoMe plane and the occlusal plane, degrees
OcP-FH	Angle between the FH plane and the occlusal plane, degrees
TAFH	Total anterior facial height, mm
PFH	Posterior Facial Height, mm
LAFH	Lower anterior facial height, mm
UAFH	Upper anterior facial height, mm
U1MP	Angle between the axis of the maxillary central incisor and the ANS-PNS plane, degrees
U1LENGTH	Length from the maxillary central incisor to the ANS-PNS plane, mm

**Table 3:** Pre- and post-expansion comparison.

Measurements	T0					T1					*p*-Value	Mean Change	95% CI Mean Change
	Mean	SD	Min	Max	95% CI	Mean	SD	Min	Max	95% CI			
SNA, degrees	80.82	3.94	69.9	88.7	79.94, 81.70	81.66	4.08	72.7	90.1	80.75, 82.56	0.001 *	0.96	0.48, 1.43
ANB, degrees	3.74	2.60	−3.9	10	3.16, 4.32	5.16	2.67	−1	11.7	4.56, 4.56	<0.000 *	1.42	1.04, 1.80
A-N perp(FH), mm	0.03	3.97	−0.85	0.91	−0.85, 0.91	0.84	3.66	0.03	1.66	0.03, 1.66	0.006 *	0.81	0.24, 1.39
SN-MP, degrees	8.11	3.52	−0.9	15.9	7.33, 8.89	8.26	3.37	0.6	15.3	7.51, 9.01	0.642	0.15	−0.50, 0.81
FH-MP (PPA), degrees	−1.16	3.40	−9.4	9.2	−1.92, −0.41	−0.98	3.38	−8.5	6.9	−1.73, −0.22	0.601	0.19	−0.51, 0.89
SNGoME, degrees	35.27	8.22	16.5	51.8	33.44, 37.10	36.25	8.61	17.6	52.1	34.33, 38.16	0.001 *	0.98	0.42, 1.54
OcP-SN, degrees	17.89	6.13	0.8	41.5	16.52, 19.25	18.24	5.70	3.2	31.3	16.97, 19.50	0.493	0.35	−0.67, 1.37
OcP-GoMe, degrees	17.38	6.57	−11.6	31.1	15.91, 18.84	18.00	6.11	1.2	32.2	16.65, 19.36	0.213	0.63	−0.37, 1.63
OcP-FH, degrees	8.63	5.38	−3.8	31.6	7.43, 9.82	9.0	5.05	−1.5	22.8	7.87, 10.12	0.464	0.37	−0.63, 1.37
TAFH, mm	117.47	9.09	77.5	140.3	115.45, 119.49	116.08	11.70	79.8	135.2	113.47, 118.68	0.132	−1.39	−3.22, 0.43
PFH, mm	76.65	8.03	52.4	99	74.86, 78.44	75.08	9.78	50.7	99.1	72.90, 77.26	0.014 *	−1.57	−2.82, −0.32
LAFH, mm	68.38	6.29	41.9	83.1	66.98, 69.78	67.62	7.73	43.2	81.9	65.90, 69.34	0.233	−0.76	−2.04, 0.52
UAFH, mm	50.90	4.17	35.9	63.9	49.97, 51.83	50.63	5.09	37.1	58.9	49.49, 51.76	0.514	−0.28	−1.10, 0.55
U1MP, degrees	108.76	7.71	91.6	124.8	107.04, 110.48	107.76	8.40	82.4	127.3	105.89, 109.63	0.072	−1.00	−2.10, 0.09
U1LENGTH, mm	29.70	2.94	20.2	35.3	29.04, 30.35	28.99	3.51	17.8	35.1	28.21, 29.77	0.033 *	−0.71	−1.36, −0.05

* Statistically significant difference.

**Table 4:** Amount of midpalatal separation in percentile groups.

Percentile Groups	Min	Max	Mean	SD	Count
1	3.3	4.8	4.10	0.55	20
2	4.81	5.7	5.22	0.27	21
3	5.71	6.81	6.26	0.32	19
4	6.81	10.17	7.89	1.58	20
Total					80

**Table 5:** Analysis of T1–T0 changes across percentile cohorts.

Variable	Group 1		Group 2		Group 3		Group 4		ANOVA	Bartlett’s Test	Kruskal–Wallis Test
	Mean	SD	Mean	SD	Mean	SD	Mean	SD			
SNA, degrees	1.33	2.05	1.06	2.17	0.99	1.80	0.04	3.48	n/a	0.02	0.66
ANB, degrees	1.63	1.84	1.29	1.84	1.54	1.68	1.24	1.49	0.86	0.78	n/a
A-N perp(FH), mm	1.74	2.53	0.28	1.83	1.52	2.97	0.23	2.54	n/a	0.02	0.04 *
SN-MP, degrees	1.07	2.17	0.32	2.11	0.88	2.69	0.72	4.11	n/a	0.01	0.22
FH-MP (PPA), degrees	0.71	3.53	0.43	2.75	1.35	2.86	0.86	3.12	0.10	0.70	n/a
SNGoME, degrees	1.21	1.54	0.80	2.10	0.93	1.83	0.98	4.01	n/a	<0.000	0.73
OcP-SN, degrees	0.11	4.15	0.38	2.16	0.18	7.28	0.93	3.84	n/a	<0.000	0.52
OcP-GoMe, degrees	1.32	4.41	0.45	2.68	0.75	6.54	0.02	3.99	n/a	<0.002	0.69
OcP-FH, degrees	0.48	4.25	1.11	2.78	0.3	6.85	1.08	3.36	n/a	<0.000	0.63
TAFH, mm	−0.25	3.76	−0.82	3.85	−1.87	8.92	−4.41	12.54	n/a	<0.000	0.87
PFH, mm	−1.21	2.37	−0.22	3.31	−1.76	6.11	−3.16	8.55	n/a	<0.000	0.68
LAFH, mm	−0.50	3.34	−0.71	2.60	−0.27	6.51	−3.04	8.39	n/a	<0.000	0.41
UAFH, mm	−0.48	2.53	−0.67	2.68	−1.32	3.88	−1.03	5.08	n/a	<0.006	0.43
U1MP, degrees	−0.35	4.42	−2.04	4.66	−2.89	3.81	−0.54	5.92	0.06	0.29	n/a
U1LENGTH, mm	−0.89	1.71	−0.06	1.67	−0.20	3.40	−2.05	3.98	n/a	<0.000	0.09

* Statistically significant difference, n/a—statistical method does not apply.

**Table 6:** Skeletal Class I and II subgroup characteristics before treatment (mean and SD).

Characteristics	Skeletal Class		
	Skeletal Class I (N = 38 (50.7%))	Skeletal Class II (N = 37 (49.3%))	Total (N = 75 (100.0%))
MEANSEP, mm	5.844 (1.68)	5.875 (1.46)	5.859 (1.57)
ANB, degrees	2.284 (1.08)	5.965 (1.43)	4.100 (2.24)
TAFH, mm	118.400 (10.96)	116.892 (6.79)	117.656 (9.12)
PPA, degrees	−1.153 (3.66)	−0.997 (3.15)	−1.076 (3.39)
PFH, mm	76.992 (8.73)	75.727 (7.45)	76.368 (8.09)
SNA, degrees	79.876 (3.91)	81.722 (3.84)	80.787 (3.96)
SNGoMe, degrees	35.253 (8.18)	36.389 (7.85)	35.813 (7.98)
SN-MP, degrees	8.366 (3.44)	8.014 (3.74)	8.192 (3.57)
U1-MP, degrees	110.011 (6.07)	106.273 (8.40)	108.167 (7.50)
OcP-SN, degrees	17.668 (4.64)	19.051 (6.95)	18.351 (5.90)
OcP-GoMe, degrees	17.576 (5.89)	17.332 (7.51)	17.456 (6.69)
OcP-FH, degrees	8.161 (4.26)	10.043 (5.78)	9.089 (5.12)
ANperpFH, mm	−0.726 (3.65)	0.749 (3.91)	0.001 (3.83)
LAFH, mm	68.424 (7.59)	68.630 (4.65)	68.525 (6.28)
UAFH, mm	51.318 (4.55)	50.722 (3.96)	51.024 (4.25)
U1-length, mm	29.521 (3.13)	30.127 (2.64)	29.820 (2.89)

**Table 7:** Skeletal Class I and II subgroup comparative analysis.

Variable	I					II					Intergroup *p*-Value
	T0		T1		*p*-Value	T0		T1		*p*-Value	
	Mean	SD	Mean	SD		Mean	SD	Mean	SD		
SNA, degrees	79.88	3.91	81.00	4.03	0.004 *	81.72	3.84	81.98	4.00	0.16	0.13
ANB, degrees	2.28	1.08	3.93	2.01	0.0052 *	5.96	1.43	6.95	1.99	0.16	0.08
A-N perp(FH), mm	−0.73	3.65	−0.084	3.39	0.15	0.75	3.91	1.62	3.21	0.027 *	0.07
SN-MP, degrees	8.37	3.44	8.33	2.87	0.92	8.01	3.73	8.77	3.59	0.12	0.22
FH-MP (PPA), degrees	−1.15	3.66	−0.70	3.48	0.36	−0.10	3.15	−0.83	3.20	0.74	0.687
SNGoME, degrees	35.25	8.18	36.03	8.28	0.01 *	36.39	7.85	37.68	8.49	0.004 *	0.69
OcP-SN, degrees	17.67	4.64	18.53	4.98	0.025 *	19.05	6.95	18.78	6.00	0.45	0.29
OcP-GoMe, degrees	17.58	5.89	17.49	5.62	0.50	17.33	7.51	18.89	6.55	0.19	0.12
OcP-FH, degrees	8.16	4.26	9.49	4.96	0.016 *	10.04	5.78	9.19	4.95	0.98	0.04 *
TAFH, mm	118.4	10.97	118.23	11.44	0.096	116.89	6.79	114.53	10.91	0.35	0.23
PFH, mm	76.99	8.73	76.64	9.29	0.36	75.73	7.45	72.98	9.77	0.001 *	0.052
LAFH, mm	68.42	7.59	68.67	7.94	0.10	68.63	4.65	66.97	6.65	0.32	0.13
UAFH, mm	51.32	4.54	51.37	4.56	0.319	50.72	3.96	50.25	5.48	0.86	0.53
U1MP, degrees	110.01	6.07	109.62	7.79	0.616	106.27	8.40	105.19	8.55	0.17	0.53
U1LENGTH, mm	29.52	3.13	29.04	3.36	0.50	30.13	2.64	29.16	3.22	0.18	0.47

* Statistically significant difference.

**Table 8:** Crude and adjusted analyses of the SNA change when using the 3D-GMP MARPE procedure in adults.

	SNA Difference		
	Coefficient	*p*-Value	95% CI
	Crude analysis		
SN-MP (T0), degrees	−0.21	0.007 *	−0.36, 0.06
	Adjusted Analysis		
SN-MP (T0), degrees	−0.24	0.114	−0.55, 0.04
Sex (male = 1, female = 0)	0.44	0.489	−0.81, 1.69
Age, years	0.19	0.56	−0.04, 0.08
MEANSEP, mm	0.35	0.52	−0.002; 0.69
ANB (T0), degrees	0.36	0.001 *	0.15; 0.57

* Statistically significant difference.

## Data Availability

The datasets used and/or analyzed during the current study are available from the corresponding author on reasonable request.

## Abbreviations

The following abbreviations are used in this manuscript:

MARPE	Miniscrew-Assisted Rapid Palatal Expansion
SARPE	Surgically Assisted Rapid Palatal Expansion
RPE	Rapid Palatal Expander
3D-GMP	Three-Dimensional-Guided Midpalatal Piezocorticotomy

## References

[1] Guest S.S., McNamara J.A., Baccetti T., Franchi L. (2010). Improving Class II malocclusion as a side-effect of rapid maxillary expansion: A prospective clinical study. Am. J. Orthod. Dentofac. Orthop..

[2] Spalj S., Zigante M., Tudor V., Öztürk T., Yağcı A., Palomo J.M. (2025). Effect of Maxillary Expansion and Protraction in Class III Children on Quality of Life, Dentofacial and Upper Airway Characteristics: A Controlled Clinical Trial. Orthod. Craniofacial Res..

[3] Maraabeh F., Shihabi R., He X., Zhan J., Zhang J., Hu L., Chen L.-L. (2025). Three-dimensional volume changes of the palatal vault and pharyngeal airway following facemask therapy and maxillary expansion: A prospective comparative study. BMC Oral Health.

[4] Brunetto D.P., Moschik C.E., Dominguez-Mompell R., Jaria E., Sant’Anna E.F., Moon W. (2022). Mini-implant assisted rapid palatal expansion (MARPE) effects on adult obstructive sleep apnea (OSA) and quality of life: A multi-center prospective controlled trial. Prog. Orthod..

[5] Maino G., Turci Y., Arreghini A., Paoletto E., Siciliani G., Lombardo L. (2018). Skeletal and dentoalveolar effects of hybrid rapid palatal expansion and facemask treatment in growing skeletal Class III patients. Am. J. Orthod. Dentofac. Orthop..

[6] Farfel V., Morea G.C., Ferreira L.M., Pereira M.D. (2022). Evaluation of Sagittal and Vertical Changes in Maxillary Dental, Skeletal, and Soft Tissue Following Surgically Assisted Rapid Maxillary Expansion: A Retrospective Longitudinal Study. J. Craniofacial Surg..

[7] Lin J.-H., Wang S., Abdullah U.A., Le A.D., Chung C.-H., Li C. (2023). Sagittal and Vertical Changes of the Maxilla after Surgically Assisted Rapid Palatal Expansion: A Systematic Review and Meta-Analysis. J. Clin. Med..

[8] Chung C.H., Woo A., Zagarinsky J., Vanarsdall R.L., Fonseca R.J. (2001). Maxillary sagittal and vertical displacement induced by surgically assisted rapid palatal expansion. Am. J. Orthod. Dentofac. Orthop..

[9] Parhiz A., Schepers S., Lambrichts I., Vrielinck L., Sun Y., Politis C. (2011). Lateral cephalometry changes after SARPE. Int. J. Oral Maxillofac. Surg..

[10] Arveda N., Migliorati M., De Mari A., Forin Valvecchi F., Schiavetti I., Annarumma F., Battista G., Aghazada H. (2025). Miniscrew-assisted slow palatal expansion with bone borne expander in adult patients: A case control study on consecutively treated patients. Angle Orthod..

[11] Walter A., Winsauer H., Crespo E., Walter D., Winsauer C., Schwärzler A., Mojal S., Arcos I., Puigdollers A. (2024). Adult maxillary expansion: CBCT evaluation of skeletal changes and determining an efficiency factor between force-controlled polycyclic slow activation and continuous rapid activation for mini-screw-assisted palatal expansion—MASPE vs. MARPE. Head Face Med..

[12] Koval S., Kolesnyk V., Chepanova D. (2025). Applications of the Novel Midpalatal Piezocorticotomy Guide for MARPE Midfacial Skeletal Expansion. J. Clin. Med..

[13] Oth O., Orellana M.F., Glineur R. (2020). The Minimally Invasive-Guided Genioplasty Technique using Piezosurgery and 3D printed surgical guide: An innovative technique. Ann. Maxillofac. Surg..

[14] AlAsseri N., Swennen G. (2018). Minimally invasive orthognathic surgery: A systematic review. Int. J. Oral Maxillofac. Surg..

[15] Choi E.-H.A., Lee K.-J., Choi S.-H., Jung H.-D., Ahn H.-J., Deguchi T., Cha J.-Y. (2023). Skeletal and dentoalveolar effects of miniscrew-assisted rapid palatal expansion based on the length of the miniscrew: A randomized clinical trial. Angle Orthod..

[16] Iodice G., Bocchino T., Casadei M., Baldi D., Robiony M. (2013). Evaluations of sagittal and vertical changes induced by surgically assisted rapid palatal expansion. J. Craniofacial Surg..

[17] Xue H., Qi Y., Ni X., Feng Y., Lin J. (2026). Efficiency of Cone-Beam Computed Tomography-Based Personalized Microimplant-Assisted Rapid Palatal Expansion in Patients With Maxillary Transverse Deficiency and Thin Palatal Bone. Int. Dent. J..

[18] Thuy P.T.H., Trang P.T., Hang P.T.T., Viet H. (2025). Clinical and cone-beam computed tomography outcomes of miniscrew-assisted rapid palatal expansion in the treatment of maxillary transverse deficiency: A prospective study. Medicine.

[19] McMullen C., Al Turkestani N.N., Ruellas A.C.O., Massaro C., Rego M.V.N.N., Yatabe M.S., Kim-Berman H., McNamara J.A., Angelieri F., Franchi L. (2022). Three-Dimensional Evaluation of Skeletal and Dental Effects of Treatment with Maxillary Skeletal Expansion. Am. J. Orthod. Dentofac. Orthop..

[20] Sayar G., Kılınç D.D. (2019). Rapid maxillary expansion outcomes according to midpalatal suture maturation levels. Prog. Orthod..

[21] Al-Mozany S.A., Dalci O., Almuzian M., Gonzalez C., Tarraf N.E., Ali Darendeliler M. (2017). A novel method for treatment of Class III malocclusion in growing patients. Prog. Orthod..

[22] Liu W., Zhou Y., Wang X., Liu D., Zhou S. (2015). Effect of maxillary protraction with alternating rapid palatal expansion and constriction vs expansion alone in maxillary retrusive patients: A single-center, randomized controlled trial. Am. J. Orthod. Dentofac. Orthop..

[23] Tarraf N.E., Dalci O., Dalci K., Altug A.T., Darendeliler M.A. (2023). A retrospective comparison of two protocols for correction of skeletal Class III malocclusion in prepubertal children: Hybrid hyrax expander with mandibular miniplates and rapid maxillary expansion with face mask. Prog. Orthod..

[24] Colak O., Paredes N.A., Elkenawy I., Torres M., Bui J., Jahangiri S., Moon W. (2020). Tomographic assessment of palatal suture opening pattern and pterygopalatine suture disarticulation in the axial plane after midfacial skeletal expansion. Prog. Orthod..

[25] Chen H., Kapetanović A., Piao Z., Xi T., Schols J.G.J.H. (2025). Stability of expansion effects following Miniscrew-assisted Rapid Palatal expansion: A prospective longitudinal cohort study. Oral Maxillofac. Surg..

[26] Shin H., Hwang C.-J., Lee K.-J., Choi Y.J., Han S.-S., Yu H.S. (2019). Predictors of midpalatal suture expansion by miniscrew-assisted rapid palatal expansion in young adults: A preliminary study. Korean J. Orthod..

[27] Koval S., Kolesnyk V., Chepanova D. (2025). Asymmetry Management During 3D-Guided Piezocorticotomy-Assisted MARPE Treatment with Direct Printed Aligners: Case Report. J. Clin. Med..

[28] Koval S., Kolesnyk V., Chepanova D. (2026). Immediate 3D Skull Changes Following 3D-Guided Midpalatal Piezocorticotomy-Assisted MARPE: Case Report. Dent. J..

[29] Pirelli P., Fiaschetti V., Fanucci E., Giancotti A., Condo’ R., Saccomanno S., Mampieri G. (2021). Cone beam CT evaluation of skeletal and nasomaxillary complex volume changes after rapid maxillary expansion in OSA children. Sleep Med..

[30] Winsauer H., Walter A., Katsaros C., Ploder O. (2021). Success and complication rate of miniscrew assisted non-surgical palatal expansion in adults—A consecutive study using a novel force-controlled polycyclic activation protocol. Head Face Med..

[31] Elshehaby M., Albelasy N.F., Elbialy M.A., Hafez A.M., Abdelnaby Y.L. (2024). Evaluation of pain intensity and airway changes in non-growing patients treated by MARPE with and without micro-osteoperforation: A randomized clinical trial. BMC Oral Health.

